# Bilateral combined anterior and posterior lenticonus in Alport’s Syndrome

**Published:** 2018

**Authors:** Uma Sharan Tiwari, Ankita Aishwarya, Rashmi Kujur

**Affiliations:** * Department of Ophthalmology, Gajra Raja Medical College, Gwalior, India

**Keywords:** Alport’s syndrome, anterior lenticonus, posterior lenticonus

## Abstract

Alport’s syndrome is an inherited disease characterized by hearing loss, progressive renal failure and ocular abnormalities like anterior lenticonus, corneal opacities, cataract, fleck retinopathies, and temporal retinal thinning.

To have anterior and posterior lenticonus in the same eye in this syndrome is a rare finding and only a few such reports are available. Hereby, we report a case of a 22-year-old male with bilateral combined anterior and posterior lenticonus with sensorineural deafness and nephritis leading to the diagnosis of Alport’s syndrome.

## Introduction

The classical Alport’s syndrome is characterized by a triad of progressive hematuria nephritis, progressive hearing loss, and ocular signs. It is an X linked disorder [**1**]. Other ocular features include corneal opacities, cataract, central, perimacular and peripheral coalescing fleck retinopathy and temporal retinal thinning [**2**]. Rarely, posterior polymorphous corneal dystrophy, macular hole, or a maculopathy impairs vision [**3**]. It is estimated that 1–4 in 100,000 children is the prevalence of posterior lenticonus [**4**]. All mutations lead to abnormalities in the cochlea, basement membrane of the glomerulus, retina, lens capsule, and cornea, which contribute to the typical phenotype of Alport’s syndrome [**5**]. Later on, cataract develops near anterior and posterior poles in areas of weakness due to tiny microscopic capsular ruptures [**6**]. The pathogenesis of Alport syndrome is explained by loss of collagen IV α3α4α5 network in basement membranes of the eye [**7**]. The importance of cataract surgery in such eyes is the careful capsulorhexis that has to be carried out as suggested by Sukhija et al. [**8**] and John et al. [**9**]. A planned cataract surgery would also avoid complications such as spontaneous rupture of the anterior lens capsule as noted previously in such eyes [**10**].

## Case report

**Fig. 1:**
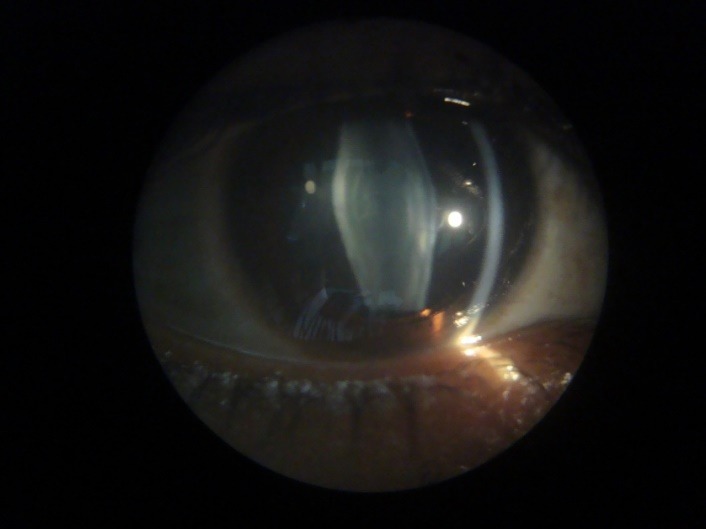
Slit lamp picture showing anterior and posterior lenticonus

**Fig. 2:**
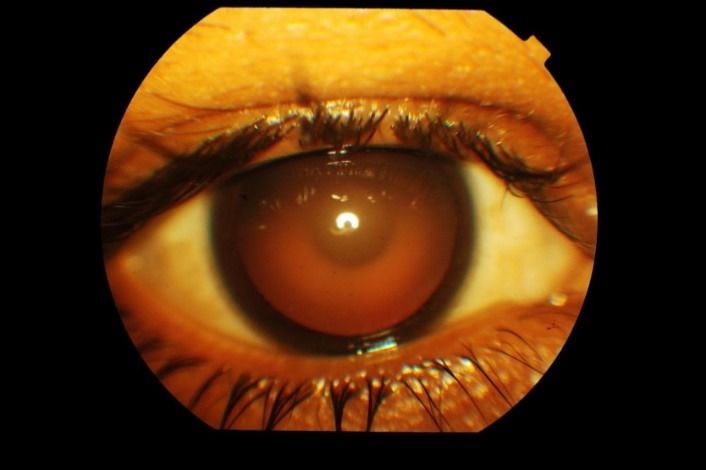
Slit lamp picture showing ‘oil droplet sign’ on retro-illumination

**Fig. 3:**
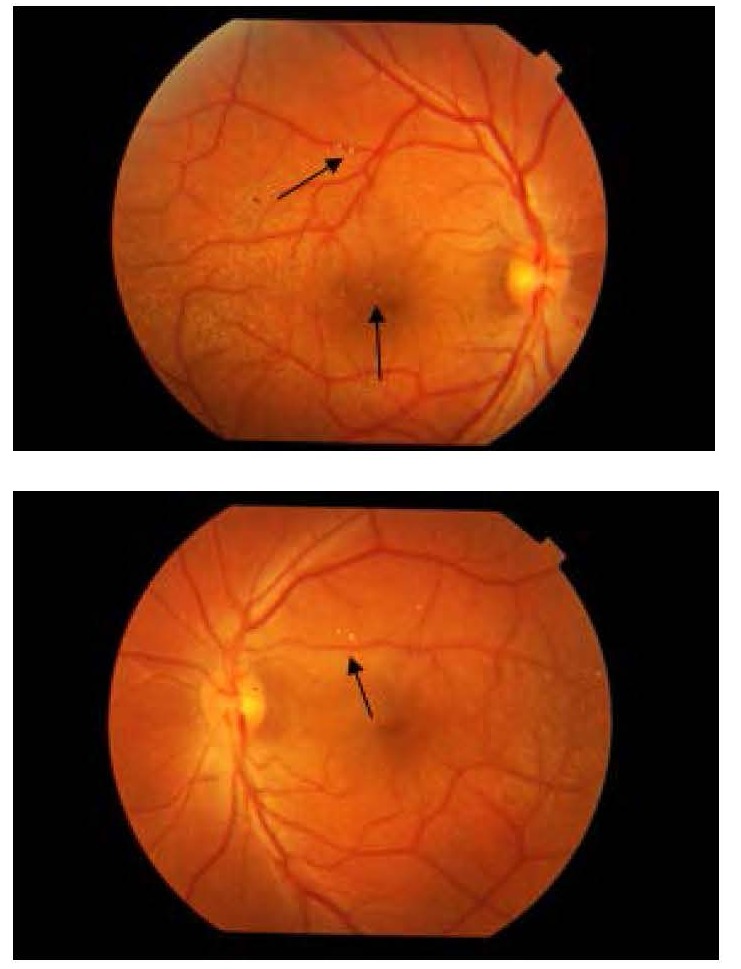
Fundus reveals bilateral retinal flecks

A 22-year-old male presented in the outpatient department with complaints of gradual diminision of vision in both eyes since eight years. His best-corrected visual acuity was 20/ 120 in both eyes. On slit lamp examination, both corneae were clear, pupillary reactions were normal and Intraocular Pressure (IOP) was 14 mm Hg in Right Eye and 12 mm Hg in Left Eye. Lens showed anterior and posterior lenticonus with anterior sub capsular lenticular opacity in both eyes (**Fig. 1**). Distant direct ophthalmoscopy revealed oil droplet reflex in both eyes (**Fig. 2**). Fundus examination revealed macular flecks in both eyes (**Fig. 3**). 

**Fig. 4:**
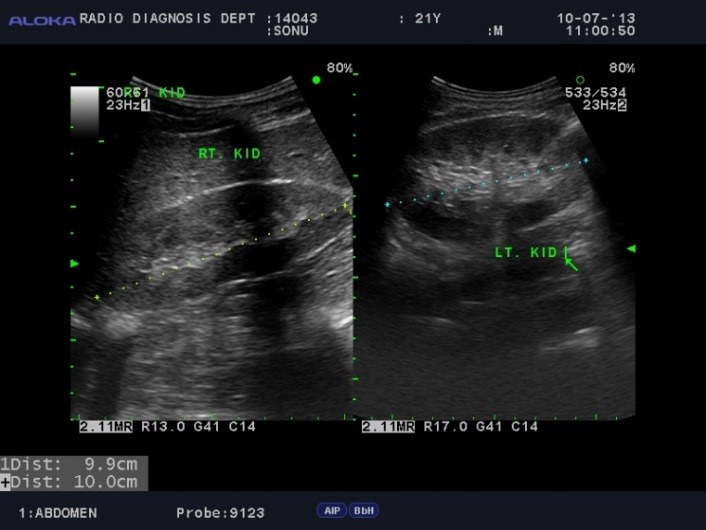
Ultrasonography of right kidney showing hypoechoic areas suggestive of nephritis

Systemic evaluation revealed sensorineural deafness bilaterally which was confirmed on audiometry. Blood investigation showed raised uric acid levels. Proteinuria was found on urinanalysis. Ultrasonography of right kidney revealed nephritis (**Fig. 4**). All these findings suggested the diagnosis of Alport’s syndrome. The patient was referred for evaluation and management by a nephrologist and an ear, nose, and throat specialist. The decision for cataract surgery was deferred for the time being and he was kept under for regular follow-up .

## Discussion

In both X-linked and autosomal recessive forms, ocular and systemic features are identical. In the autosomal dominant form of Alport syndrome which is rare entity, have only retinopathy and cataracts as ocular abnormalities[**5**]. COL4A5, COL4A3, and COL4A4 genes which are α-5-chain of the type IV collagen gene have been mapped as defects in the X-linked form of Alport syndrome . All these mutations generally lead to abnormalities in cochlea, the basement membrane of the glomerulus, cornea, retina, lens capsule, all of which eventually contribute to the phenotype of Alport syndrome [**5**]. 

Posterior lenticonus, was once considered as an isolated manifestation is found to be the part of disease, being reported frequently in association with Alport syndrome [**11**]. Bilateral combined anterior and posterior lenticonus in Classic Alport’s syndrome is a rare finding and has been described in few reports [**12**]. 

Cataract in these patients can occur either as a component of the disease or as a side effect of oral steroids following renal transplant, along with a fragile capsule makes cataract surgery more challenging [**11**]. Cataract surgery in such patients can be planned for rehabilitation of the patient but care should be taken while performing capsulorhexis. A pre-existing posterior capsular defect can exist for which a posterior capsulorhexis may be required.

## Conclusion 

The ocular manifestations of Alport’s syndrome should be recognized as early as possible. An early diagnosis of Alport syndrome can be life saving as it decreases the risk of early renal failure.

**Declaration of patient consent**

We certify that we have obtained all appropriate consents from the patient. The patient has given consent for her images and other clinical information to be reported in the journal. The patient understands that her name and initials will not be published and due efforts will be made to conceal her identity, but anonymity cannot be guaranteed.

**Acknowledgment**

None.

**Financial support and sponsorship **

None.

**Conflicts of interest**

None.
